# Diagnostic Accuracy of FDG‐PET‐CT in Dementia: Assessing False Negatives in Clinical Practice

**DOI:** 10.1002/alz70862_109960

**Published:** 2025-12-23

**Authors:** Autumn Shoebridge, Beili Shao, Abigail Rebecca Lee, Hannah Sargisson, Elizabeta Mukaetova‐Ladinska, Akram A. Hosseini

**Affiliations:** ^1^ University of Nottingham, Nottingham, Nottinghamshire UK; ^2^ School of Medicine, University of Nottingham, Nottingham, Nottinghamshire UK; ^3^ Academic Neurology, Nottingham University Hospitals NHS Trust, Queen's Medical Centre, Nottingham, Nottinghamshire UK; ^4^ School of Psychology, University of Leicester, Leicester, Leicestershire UK

## Abstract

**Background:**

Diagnosing dementia can be challenging due to its multiple causes and overlapping presentations, especially in the early stages when cognitive impairment is mild, leading to misdiagnosis in clinical practice. Previous research assesses the use of neuroimaging for the diagnosis of Alzheimer’s disease (AD). However, it is unclear whether these findings are replicable in real‐world clinical practice and in non‐AD dementia.

**Method:**

To explore the diagnostic accuracy of fluorodeoxyglucose (FDG)‐positron emission tomography (PET) computed tomography (CT) of the brain by assessing the proportion of false negatives for different dementia diagnoses. FDG‐PET CT scans and neuropsychological test scores from 155 participants aged between 36‐82 (mean 62 years; 56% male) with Alzheimer’s disease and related dementia (ADRD) were included in the analysis. Their clinical diagnosis of study participants was based on clinical and neuroradiological assessments and included longitudinal neurocognitive tests, Magnetic Resonance Imaging (MRI) of the brain, FDG‐PET‐CT of the brain, and the examination of cerebrospinal fluid (CSF) for amyloidβ, total tau and 181‐Phsophorlyated‐tau measurements. The data was obtained from CogNID (Cognitive and NeuroImaging in neurodegenerative Disorders, IRAS: 250525, ClinicalTrials.gov Identifier: NCT03861884), a prospective study inviting patients who are referred to a Nottingham tertiary referral centre from the Memory clinics in the East Midlands, and Cognitive Clinics at the Queen’s Medical Centre.

**Result:**

The proportion of false negatives from FDG‐PET ranged from 11‐55%. The lowest proportion of false negatives was found in the Frontotemporal dementia diagnosis group, and the highest proportion of false negatives was found in the other neurodegeneration group. In early‐onset dementia patients (*N* = 112), the proportion of false negatives ranged from 15‐60%.

**Conclusion:**

FDG‐PET‐CT of the brain produces a notable proportion of false negatives in dementia workups, suggesting limitations in sensitivity. These findings indicate that FDG‐PET‐CT alone may not be sufficiently reliable for diagnostic use in clinical practice and should be complemented by additional diagnostic methods.

